# Challenges in warfarin versus DOACs for elderly atrial fibrillation: A critique of Takagi and Ueda's study

**DOI:** 10.1002/joa3.70114

**Published:** 2025-06-23

**Authors:** Sanjana, Tazeen Saeed Ali, Ashfaq Ahmad, Javed Iqbal, Iqra Khan

**Affiliations:** ^1^ Medicine Department Liaquat University of Medical Health Science Jamshoro Sindh Pakistan; ^2^ School of Nursing and Midwifery and Department of Community Health Sciences The Agha Khan University Karachi Pakistan; ^3^ Medicine Department Gomal Medical College D.I. Khan Dera Ismail Khan Pakistan; ^4^ Nursing Department Hamad Medical Corporation Doha Qatar; ^5^ Medicine Department Services Institute of Medical Sciences Lahore Pakistan

To The Editor,

The article titled “Factors related to the choice of warfarin for treating newly diagnosed non‐valvular atrial fibrillation is associated with safety outcomes during anticoagulation: A new‐user, active‐comparator, retrospective cohort study”^1^ by the authors Yoshiko Takagi and Shinichiro Ueda clearly outlines the study's comparison of warfarin and direct oral anticoagulants (DOACs) for anticoagulation therapy in nonvalvular atrial fibrillation (NVAF).

I truly appreciate the valuable insights into the safe and unsafe use of warfarin and direct oral anticoagulants provided by this study. Still, a few knowledge gaps affect the generalizability and applicability of the well‐defined retrospective cohort study. The article reported the outcomes after giving trials of warfarin and direct oral anticoagulants in patients with nonvalvular atrial fibrillation and found that warfarin is the first choice of physicians over direct oral anticoagulants for old age patients with nonvalvular atrial fibrillation considering their chronic health issues. Hence, warfarin is also associated with a high risk of bleeding as compared to direct oral anticoagulants, which leads to a low risk of bleeding. Still, it remains a challenge whether to prescribe warfarin or direct oral anticoagulants for high‐risk patients.

I want to highlight the robust study design, including a large sample size of 2979 patients and using propensity scores ideally to evaluate the effectiveness and safety of warfarin and direct oral anticoagulants. A great approach to organizing an article's writing style is to reflect a broader audience to get access to reading and use it as a citation in their studies. The findings of the study indicate the potential risks for prescribing warfarin and direct oral anticoagulants in older age patients and make it feasible to use or not use them by justifying the outcomes according to the health issues of patients. The authors have comprehensively provided the section on limitations, declared the potential biases, and stated the research domains for future studies.

“Despite the study's strengths and limitations,” I sense that the authors could work on some weak areas to strengthen the findings. For instance, the authors did not lay out the information about medical adherence, which is a significant drawback in this study to prove the efficiency of the final results. A population‐based cohort study distinctly stated that availing medical adherence as a potent tool for their study aided in acquiring the profound desired results for comparing different anticoagulants.^2^ For clinical trials, it is very mandatory to keep an eye on the drugs properly being administered to the patients participating in the study because this is how the study gets its final unbiased results; otherwise, patients can skip the medications, which proves to be the most significant disadvantage of highly comprehensive studies. The characteristics of the healthcare providers, such as their experience or specialty, can potentially alter the outcomes because the choice of anticoagulant therapy prescribing will be wholly dependent on them.^3^ The more healthcare providers experienced, the more they would know which drug is effective in the patients with nonvalvular atrial fibrillation, keeping in account the other old age‐related health issues. The authors did not mention this factor in the analysis, as this factor should be discussed for the authenticity of precise results.

Regarding the potential bias, we know it is a drawback of a retrospective study. The authors also have mentioned it in the limitation section but have not discussed how it can be abolished by various methods, such as checking the sensitivity analysis of the article. By checking sensitivity analysis through common techniques like tornado diagrams and spider plots using different software, for example, R, Python, and Excel, one could quantify the uncertainty in the final results and help establish the robustness of findings.^4^

In conclusion, a retrospective study suggests a link between the factors influencing warfarin selection for new‐onset nonvalvular atrial fibrillation and subsequent safety outcomes during anticoagulation. It falls short in several key areas. Ultimately, addressing the issues that I highlighted is crucial for the better outcomes of the research and making it high yielding for clinical interventions. In the final analysis, the authors should implement the possible changes to create a powerful study. As we move forward, it is essential to prioritize the safety of anticoagulant therapy in nonvalvular patients to ensure good health.

## FUNDING INFORMATION

None.

## CONFLICT OF INTEREST STATEMENT

The authors declare no conflict of interest.

## ETHICS STATEMENT

The authors declare no disclosures.

## Data Availability

This letter discusses publicly available data from the cited original publication. No new data were generated or analyzed for its preparation.
